# Identification of a novel functional miR-143-5p recognition element in the Cystic Fibrosis Transmembrane Conductance Regulator 3′UTR

**DOI:** 10.3934/genet.2018.1.53

**Published:** 2018-02-23

**Authors:** Chiara De Santi, Sucharitha Gadi, Agnieszka Swiatecka-Urban, Catherine M. Greene

**Affiliations:** 1 Lung Biology Group, Department of Clinical Microbiology, RCSI Education & Research Centre, Beaumont Hospital, Dublin 9, Ireland; 2 Children's Hospital of Pittsburgh of UPMC, Department of Cell Biology, University of Pittsburgh School of Medicine Pittsburgh, PA, USA

**Keywords:** microRNA, cystic fibrosis, CFTR, qRT-PCR, luciferase assay

## Abstract

MicroRNAs (miRNAs) are small non-coding RNAs involved in regulation of gene expression. They bind in a sequence-specific manner to miRNA recognition elements (MREs) located in the 3′ untranslated region (UTR) of target mRNAs and prevent mRNA translation. MiRNA expression is dysregulated in cystic fibrosis (CF), affecting several biological processes including ion conductance in the epithelial cells of the lung. We previously reported that miR-143 is up-regulated in CF bronchial brushings compared to non-CF. Here we identified two predicted binding sites for miR-143-5p (starting at residues 558 and 644) on the *CFTR* mRNA, and aimed to assess whether *CFTR* is a true molecular target of miR-143-5p. Expression of miR-143-5p was found to be up-regulated in a panel of CF vs non-CF cell lines (1.7-fold, P = 0.0165), and its levels were increased *in vitro* after 20 hours treatment with bronchoalveolar lavage fluid from CF patients compared to vehicle-treated cells (3.3-fold, P = 0.0319). Luciferase assays were performed to elucidate direct miRNA::target interactions and showed that miR-143-5p significantly decreased the reporter activity when carrying the wild-type full length sequence of *CFTR* 3′UTR (minus 15%, P = 0.005). This repression was rescued by the disruption of the first, but not the second, predicted MRE, suggesting that the residue starting at 558 was the actual active binding site. In conclusion, we here showed that miR-143-5p modestly but significantly inhibits CFTR, improving the knowledge on functional MREs within the *CFTR* 3′UTR. This could lead to the development of novel therapeutic strategies where miRNA-mediated CFTR repression is blocked thereby possibly increasing the efficacy of the currently available CFTR modulators.

## 1. Introduction

MicroRNAs (miRNAs) are small non-coding RNA molecules discovered in the early nineties in nematodes [1] and repeatedly shown to play a crucial role in regulation of gene expression in various animal models [2] and human diseases [3]–[5]. MiRNAs mainly exert their function as negative regulators by binding in a sequence-specific manner to miRNA recognition elements (MREs) particularly in the 3′ untranslated region (UTR) of a target mRNA and thus preventing mRNA translation [6]. In animals, the miRNA::mRNA base-pairing is usually limited to the so-called “seed region”, i.e., the miRNA sequence (often between the 2^nd^ and the 8^th^ nucleotide) that provides most of the pairing specificity [7]. The mRNA region complementary to the miRNA seed is called “seed match” and its genetic alteration could have important phenotypic consequences in terms of risk of developing cancer [8], myocardial infarction [9] and chemo-resistance [10]. The identification of the miRNA:mRNA pairing has been widely addressed using publically available bioinformatics tools followed by experimental validation [11].

We and other research groups have previously identified several miRNAs targeting *CFTR* (Cystic Fibrosis Transmembrane conductance Regulator) mRNA that encodes for an ATP-regulated anion channel present on the apical surface of epithelial cells [12]–[18]. Mutations in the *CFTR* gene are the primary cause of cystic fibrosis (CF), a life-threatening disorder characterised by chronic airway inflammation and infection. CF is the most common lethal genetic disease among the Caucasian population, with 1 in 3000 newborns found to be affected by CF worldwide [19]. Although about 2000 *CFTR* mutations have been identified, only 281 have been confirmed as CF-causing to date, including ΔF508, G542X, G551D and W1282X [20].

MiRNA expression has been reported to be altered in CF patients, thereby affecting several biological processes such as ion conductance, endoplasmic reticulum stress, inflammation and infection [21]. In particular, we and others have shown that several miRNAs targeting CFTR were up-regulated in CF bronchial brushings [15] and CF primary cultures [17]. Although different experimental situations were investigated, such as the use of different cell lines and examining response to cigarette smoke, miR-101, miR-145, miR-223, miR-494 and miR-509-3p have been repeatedly implicated in many studies, strongly highlighting their roles in regulating CFTR expression [12]–[18]. In addition to these miRNAs, data from our previously published miRNA profiling indicate miR-143 as a potential regulator of *CFTR* mRNA. Its levels are increased in CF vs. non-CF bronchial brushings [22], and there are two potential miR-143-5p binding sites in the *CFTR* 3′UTR. In this article, we confirmed the up-regulation of miR-143-5p in CF cell lines and tested the hypothesis that miR-143-5p regulates CFTR expression via binding sites on *CFTR* 3′UTR.

## 2. Materials and methods

### 2.1. Cell culture and treatments

Non-CF (16HBE14o^−^) and CF (CFBE41o^−^, genotype ΔF508/ΔF508) human bronchial epithelial cells were kindly donated by D. Gruenert (California Pacific Medical Centre Research Institute, San Francisco, CA, USA) and maintained in MEM+Glutamax (Gibco) with 10% fetal calf serum (FCS; Gibco) and 1% penicillin–streptomycin (Pen-Strep; Gibco). IB3 cells are ΔF508/W1282X CF bronchial epithelial cells, S9 are their isogenic non-CF counterpart and both were obtained from Pamela Zeitlin (Johns Hopkins Children's Centre, Baltimore, MD, USA) and cultured in LHC-8 medium (Gibco) with 5% FCS and 1% Pen–Strep. Non-CF (Nuli-1) and CF (Cufi-1, genotype ΔF508/ΔF508) human bronchial epithelial cells were received as a gift from Prof Zabner (University of Iowa, USA) and grown in Bronchial Epithelium Basal Medium (BEBM, Lonza) supplemented with BEGM SingleQuots Kit (Lonza, containing epidermal growth factor, hydrocortisone, bovine pituitary extract, transferrin, bovine insulin, triiodothyronine, epinephrine, retinoic acid), gentamicin (Gibco, 10 µg/mL), and amphotericin B (Gibco, 1.25 µg/mL).

For cell treatments, bronchoalveolar lavage (BAL) fluid was recovered from CF patients (n = 6, mean ± SD age 28 ± 12 years, male:female 3:3) as previously reported in [23],[24] and pooled for experiments. CF individuals were confirmed by sweat testing and/or genotyping and are listed in online Supplementary Table S1. After 24-h serum starvation, CF cells (CFBE41o^−^, IB3 and Cufi-1) seeded in 24-well plates (100,000 cells/well) were treated with 1% CF BAL fluid (10 µL/mL) or 1% saline solution (0.9% w/v NaCl) for a further 20 hours in medium containing 1% FCS. RNA was extracted for miRNA expression analysis performed with quantitative real-time PCR (qRT-PCR). Three independent experiments were carried out in duplicate for each cell line.

For luciferase assays, the human embryonic kidney cell line HEK293 was obtained from the European Collection of Cell Cultures (Salisbury, UK) and maintained in DMEM (Lonza) with 10% FCS and 1% Pen-Strep.

All the cell lines were maintained in a humidified incubator at 37 °C in 5% CO_2_.

### 2.2. miRNA qRT-PCR

RNA from CF and non-CF cell lines (n = 6 for each cell line) was purified by using Tri-Reagent (Sigma Aldrich) according to the manufacturer's instructions and its purity and concentration were measured using a Nanodrop 8000 (ThermoFisher Scientific). Tri-Reagent was also used to extract RNA from CF cells treated for 20 hours with 1% CF BAL fluid/1% saline solution. The TaqMan® MicroRNA Reverse Transcription Kit (ThermoFisher Scientific) was employed for reverse transcription using stem-loop specific miRNA primers starting from 100 ng of total RNA. Expression of miR-143-5p (assay ID: 002146) was measured using a pre-designed TaqMan MicroRNA assay (ThermoFisher Scientific) on the LC480 LightCycler (Roche). In each well, 1.33 µL of cDNA were added to the reaction mixture which consisted of 7.67 µL of deionized H_2_O, 1 µL of specific TaqMan Assay probe and primer mixture, and 10 µL of TaqMan® Universal Master Mix II NO UNG (ThermoFisher Scientific). Each sample was run in triplicate with the following the thermocycling conditions: 10 min at 95 °C followed by 40 cycles of 15 s at 95 °C and 60 s at 60 °C. Expression of miR-143-5p relative to U6 snRNA (assay ID: 001973) was determined using the 2^(−ΔΔCt)^ method [25].

### 2.3. Plasmid construction and dual-luciferase assay

*In silico* prediction analyses for miR-143-5p binding sites on the *CFTR* 3′UTR were performed with TargetScan 7.1 (release June 2016, [26]), miRanda (release August 2010, [27]) and RNA22 [28] software.

The full length of the human *CFTR* 3′UTR (1556 bp) was amplified by Q5 high-fidelity DNA polymerase (NEB) from DNA extracted from a healthy individual without CF. The PCR amplicon was subsequently purified with Wizard® SV Gel and PCR Clean-Up System (Promega) and cloned into the *Xho*I site of the pmirGLO vector (Promega) using CloneEZ PCR Cloning Kit (GenScript), downstream to the *luciferase* reporter gene sequence. The plasmid carrying the wild-type (wt) sequence of *CFTR* 3′UTR is now on referred as “pmir_CFTR_wt”. Plasmids were isolated from bacterial cultures with Plasmid Midi Kit (Qiagen). Subsequent mutagenesis reactions aimed to disrupt the miR-143-5p binding sites within the 3′UTR sequence were performed using with QuikChange II Site-Directed Mutagenesis Kits (Agilent) and screened with allele-specific oligonucleotide PCR (ASO-PCR). Three mutant plasmids were generated: two single mutants in which the first (starting at residue 558 of *CFTR* 3′UTR, wt: cacaggaaccacaagACTGCACa, mutant: cacaggaaccacaagAC**GAA**ACa) or the second (starting at residue 644 of *CFTR* 3′UTR, wt: atcagggttagtATTGTCc, mutant: atcagggttagtAT**CAC**Cc) miR-143-5p site is mutated and one in which both miR-143-5p sites are mutated (a double mutant). The three plasmids are now on referred as “pmir_CFTR_mut1”, “pmir_CFTR_mut2” and “pmir_CFTR_mut1+2”. The fidelity of the resulting constructs was confirmed by sequencing, using the pmirGLO external primers. The sequence of cloning, mutagenesis and sequencing primers is reported in Supplementary Table S2.

In luciferase assay experiments, HEK293 cells were seeded in a 96-wells plate at a final density of 10,000 cells/well and incubated for 24 hours. Cells were then co-transfected with 100 ng of pmir_CFTR_wt/mut1/mut2/mut1+2 and 100 nM of miR-143-5p mimic or negative control mimic (ThermoFisher Scientific, assay ID MC12540 and 4464058, respectively). Transfection mixes were prepared in Optimem reduced-serum media (Gibco) using Genejuice and Ribojuice (Novagen) as transfection reagents for plasmid DNA and miRNA mimics, respectively. Luciferase activity was assessed at 24 hours after transfection using Dual-Luciferase Reporter Assay (Promega) according to the manufacturer's instructions. Briefly, cells were washed with PBS and harvested with 20 µL of passive lysis buffer (PLB 1x). Firefly luciferase activity was measured by adding LAR II buffer to the cell lysates in a white 96-wells plate. Then, Stop & Glo reagent was added and the *Renilla* luciferase activity, used as normaliser for cell number and transfection efficiency, was assessed. RLU (relative luciferase units) expressed as mean value of the firefly luciferase/*Renilla* luciferase ratio of three independent experiments performed in triplicate were used for statistical analyses.

### 2.4. Statistical analysis

All statistical analyses were performed using GraphPad PRISM 7.0 (San Diego, CA). Results are expressed as the mean ± standard error of the mean (SEM) and were compared by Student's *t* test or ANOVA, as appropriate. Differences were considered statistically significant when P ≤ 0.05.

## 3. Results

### 3.1. miR-143-5p is overexpressed in CF airway epithelial cells and the levels increase in response to treatment with the CF BAL fluid

**Figure 1.**
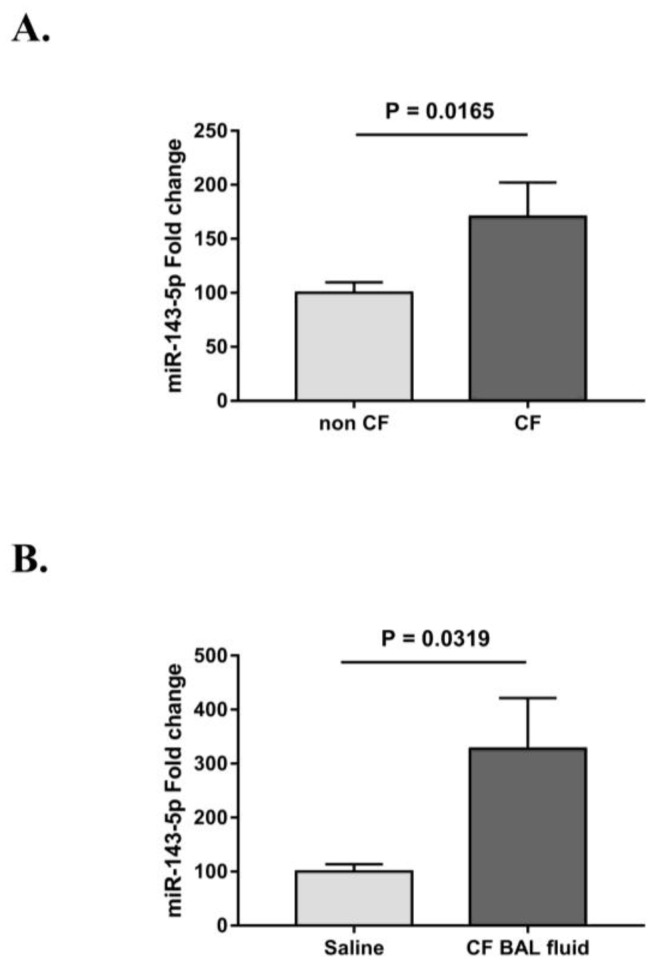
Relative expression of miR-143-5p in (A) CF (CFBE41o^−^, IB3 and Cufi-1) vs non-CF (16HBE14o^−^, S9 and Nuli-1) cells (n = 6 for each cell line) and (B) CF cell lines treated with CF BAL fluid vs vehicle control (saline solution) for 20 h in three independent experiments (in duplicate). Non-CF cells or control-treated samples are reported as reference and set at 100%. Data are presented as mean ± SEM and were compared by Student's *t* test.

We previously reported that levels of miR-143 are increased 2.3-fold in CF vs. non-CF bronchial brushings (p = 0.049, n = 5 each) [22]. Here, we aim to determine whether matched immortalised CF and non-CF bronchial epithelial cell lines represent useful models to recapitulate our *in vivo* findings. We first compared the basal expression of miR-143-5p in matched pairs of CF vs non–CF cell lines. MiR-143-5p levels were trending upwards in each of the three CF cell lines versus their non-CF counterparts (Supplementary Figure S1). Overall miR-143-5p expression in CF versus non-CF cells was significantly increased by 1.7-fold (Figure 1A).

Moreover, in order to assess whether the highly inflamed environment of CF lungs affected miR-143-5p expression *in vitro*, CF cells were treated for 20 hours with CF BAL fluid or saline solution (as control vehicle) prior to RNA extraction in three independent experiments. We observed an increasing trend for miR-143-5p expression in all cell lines following treatment with CF BAL fluid (Supplementary Figure S2), and overall the fold-increase was 3.3 (Figure 1B).

### 3.2. miR-143-5p mediates repression of the CFTR 3′UTR via one binding site

**Figure 2.**
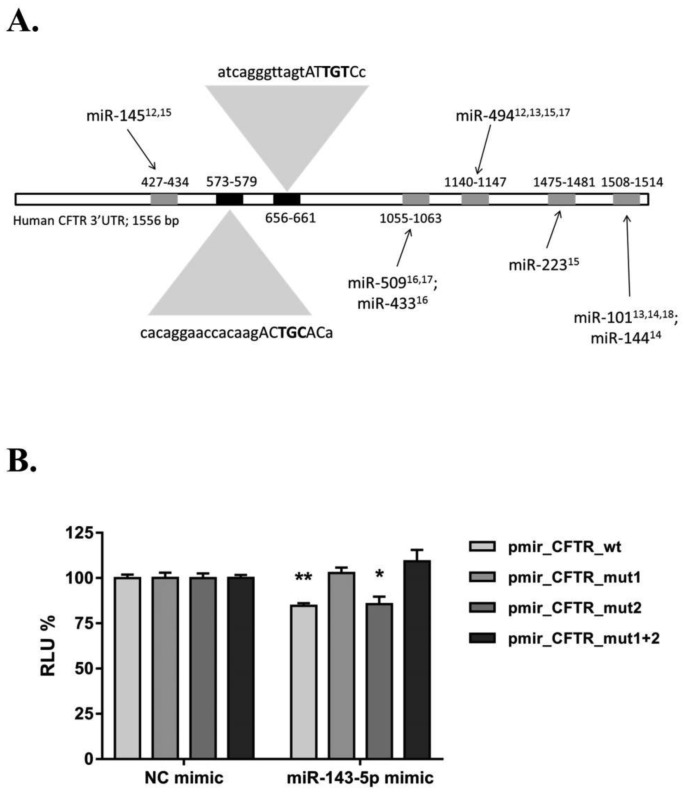
(A) visual map of MREs in the *CFTR* 3′UTR: previously published MREs with appropriate references are reported in grey, the miR-143-5p predicted binding sites are coloured in black and their sequence (capital letters represent the seed match, with bases in bold being subsequently mutated for the luciferase assay) is also reported. The position of the seed match for all the MREs within CFTR 3′UTR is also shown. (B) Luciferase activity expressed as RLU in HEK293 cells in three independent experiments (in triplicate). Samples cotransfected with NC mimic are reported as reference and set at 100%. Data are presented as mean ± SEM and were compared by two-way ANOVA (Sidak's multiple comparisons test *P < 0.05, **P < 0.01). MiR-143-5p mimic significantly decreased luciferase activity in pmit_CFTR_wt and pmir_CFTR_mut2, while it was ineffective when cotransfected with pmir_CFTR_mut1 and pmir_CFTR_mut1 + 2.

In order to assess the ability of miR-143-5p to regulate *CFTR* expression, we employed publically available software to identify putative MREs in the *CFTR* 3′UTR. TargetScan 7.1 and miRanda identified the same binding site, starting at residue 558 (seed match 573–579) of the *CFTR* 3′UTR, while RNA22 predicted a second binding site starting at residue 644 (seed match 656-661), as shown in Figure 2A. Site-directed mutagenesis was successfully performed in order to disrupt these MREs and an example of post-mutagenesis screening with ASO-PCR is provided in Supplementary Figure 3.

For luciferase assays, HEK293 cells were transiently transfected with a luciferase reporter plasmid containing the full length of wild type *CFTR* 3′UTR or with the same plasmid where the MREs were mutated to assess whether *CFTR* is a true target of miR-143-5p. Transfection efficiency was normalised between wells by measuring the activity of the internal control reporter *Renilla* and by using RLU values in the statistical analyses. Cotransfection with miR-143-5p mimic resulted in a significant decrease (minus (−)15%) of luciferase activity, expressed as RLU, in the pmir_CFTR_wt plasmid when compared to the negative control (NC) mimic cotransfected wells (Figure 2B). Interestingly, mutations of the first but not the second MRE abolished the inhibitory effect of miR-143-5p on the luciferase activity. When both the sites were mutated, miR-143-5p was not able to decrease luciferase activity, suggesting that the disruption of the first MRE was necessary and sufficient to rescue the miRNA-mediated repression of the reporter activity.

## 4. Conclusions

In this paper, we observed increased levels of miR-143-5p in CF versus non-CF cell lines and we proved that it directly targets *CFTR* 3′UTR through a binding site starting at residue 558. In detail, unstimulated CF cell lines expressed significantly higher basal levels of miR-143-5p when compared with their non-CF counterparts, and the increase was even more evident when CF cell were treated with CF BAL fluid, suggesting that the inflamed environment of CF lungs could trigger the upregulation of miR-143-5p (Figure 1). The detailed mechanism is currently unknown and beyond the scope of this study. We have previously shown that proinflammatory mediators such as *Pseudomonas* conditioned media or IL-1β (potentially present in the CF BAL fluid) could increase expression of CFTR-targeting miRNAs [15]. Similarly, increased levels of miR-509-3p and miR-494 were observed by Ramachandran et al. when treating non-CF airway epithelia with the proinflammatory cytokines TNF-α and IL-1β [17]. Overall, these findings suggest that CF airway epithelial cells, especially when treated with *ex vivo* CF stimuli, can recapitulate our *in vivo* findings about the upregulation of miR-143.

Luciferase reporter assay is considered the most reliable technique to identify functional MREs within 3′UTRs [29]. Here we observed a decrease in luciferase activity after transfection of miR-143-5p mimic from the reporter plasmid carrying the wild-type *CFTR* 3′UTR suggesting that this miRNA directly regulates *CFTR*. The site-directed mutagenesis is necessary to determine whether the predicted binding sites are functional, and our results showed that the MRE starting at residue 558 was actually active, as its disruption led to a rescue of the reporter activity inhibition by miR-143-5p mimic. According to our data, the second site (starting at residue 644) was not active, showing once again that bioinformatic target prediction can give false positive results, and experimental validation is ultimately required to truly determine miRNA::mRNA binding and biological function.

MiR-143 is located in the 5q32 chromosomal region and it has been previously identified as tumour suppressor in several types of cancer [30]. In particular, miR-143-5p inhibits cell proliferation, migration and invasion in gallbladder cancer targeting *HIF-1α*, ultimately preventing the epithelial-to-mesenchymal transition [31]. Similar anti-tumorigenic properties were observed in gastric cancer where miR-143-5p induced apoptosis directly targeting *COX2* [32].

Beyond the importance of describing the regulators of CFTR expression, identifying cognate miRNAs could have valuable therapeutic effects. Over the past decades, therapies for CF aimed to cure the symptoms of the disease rather than its primary cause. Ivacaftor (VX-770) has been approved for marketing in Europe and the USA for CF patients carrying the G551D mutation (and the other mutations recently added [33]) as the first drug targeting the actual CFTR dysfunction [34]. In order to correct the CFTR misfolding due to ΔF508 mutations, a new molecule called lumacaftor (VX-809) has been developed but its administration in clinical trials did not provide significant benefits for ΔF508/ΔF508 CF patients [35]. Even the combination of VX-770 and VX-809 led only to modest improvement of the respiratory function in phase II [36] and III [37] clinical trials in ΔF508/ΔF508 CF patients, suggesting an urgent need of boosting the activity of CFTR modulators. As miRNAs act upstream of correctors and potentiators, blocking miRNA-mediated CFTR repression may increase the efficacy of CFTR modulators in ΔF508/ΔF508 patients. Viart and collaborators treated nasal epithelial cells from CF patients with miRNA-binding blocker oligonucleotides (MBBOs) specifically designed to bind the MREs of several CFTR-targeting miRNAs (including miR-101 and miR-145) and found a significant increase in CFTR expression and function [18]. Very recently, Zarrilli and co-authors developed peptide nuleic acids (PNAs) in order to prevent miR-509-3p binding from its MREs on *CFTR* mRNA and showed that a PNA was able to rescue the miRNA-mediated inhibition in luciferase assay [38]. Altogether, these studies suggest that interfering with CFTR-targeting miRNAs represents a promising therapeutic strategy, although the combination with existing correctors or potentiators has not been explored yet.

Although further studies are necessary to establish whether miR-143-5p represents a valuable miRNA for future therapeutic studies in CF, here we suggest another functional MRE in the *CFTR* 3′UTR worthy to be investigated. The mapping of MREs on the *CFTR* 3′UTR could help to design combination therapies where more than one miRNA is blocked and thus a greater effect on CFTR expression could be achieved. This knowledge would not be limited to CF, but it could also potentially be applied to any disease that shows an acquired CFTR dysfunction and where modulation of CFTR could be beneficial [39],[40].
