# Supplementary material for: Identification of a novel functional miR-143-5p recognition element in the Cystic Fibrosis Transmembrane Conductance Regulator 3′UTR
Source: AIMS Genet. 2018 Feb 23;5(1):53–62. doi: 10.3934/genet.2018.1.53 (PMC6690249; doi:10.3934/genet.2018.1.53)
Supplement: Supplementary file 1 [file genetics-05-01-053-s01.pdf]

AIMS Genetics, 5(1): 53–62.
DOI: 10.3934/genet.2018.1.53
Received: 01 September 2017
Accepted: 08 February 2018
Published: 23 February 2018

http://www.aimspress.com/journal/Genetics

Research article

Identification of a novel functional miR-143-5p recognition element in the Cystic Fibrosis Transmembrane Conductance Regulator 3'UTR

Chiara De Santi[1,]*, Sucharitha Gadi[1], Agnieszka Swiatecka-Urban[2] and Catherine M. Greene[1]

[1] Lung Biology Group, Department of Clinical Microbiology, RCSI Education & Research Centre, Beaumont Hospital, Dublin 9, Ireland
[2] Children's Hospital of Pittsburgh of UPMC, Department of Cell Biology, University of Pittsburgh School of Medicine Pittsburgh, PA, USA

* **Correspondence:** Email: chiaradesanti@rcsi.ie; Tel: +35318093800.

Supplementary

Table S1. Details of CF patients included in the manuscript for the bronchoalveolar lavage (BAL) fluid collection.

Patient Number	Gender	Genotype	Age (years)*
1	F	ΔF508/W361R	51
2	F	G551D/G542X	26
3	F	unknown	30
4	M	ΔF508/E60X	20
5	M	ΔF508/ΔF508	16
6	M	ΔF508/E60X	24

*Age at the time of the BAL fluid collection is reported

Table S2. Primers names and sequences employed in this study.

Name	Sequence
Cloning primers	
CLON_CFTR_F	AACGAGCTCGCTAGC**CTCGAG**agagcagcataaatgttgac
CLON_CFTR_R	CAGGTCGACTCTAGA**CTCGAG**ttcacacaaatgtatgtatggatt
Mutagenesis primers	
CFTR_MUT_1	agaataccacaggaaccacaagac**GAA**acatcaaaatatgccccattcaac
CFTR_MUT_2	acttccagatcctggaaatcagggttagtat**CAC**ccaggtctaccaaaaa
ASO-PCR primers	
ASO_wt_1	cacaggaaccacaagac**TGC**
ASO_mut_1	cacaggaaccacaagac**GAA**
ASO_wt_2	cctggaaatcagggttagtat**TGT**
ASO_mut_2	cctggaaatcagggttagtat**CAC**
Sequencing primers	
pmir_seq_F	gtggtgttgtgttcgtggac
pmir_seq_R	cagccaactcagcttccttt

Cloning primers: according to the manufacturer's instructions for the CloneEZ PCR cloning kit, primers covered a 15-base sequence add-on at the 5'-end (capital letters, underlined), an optional restriction site in the middle (capital letter, in bold), and the insert-specific sequence at the 3'-end. CLON_CFTR_F and CLON_CFTR_R primers were designed to amplify the full length of CFTR 3'UTR (1556 bp). Mutagenesis primers: the mutant nucleotides are reported in capital letter, bold. ASO-PCR primers: Allele-Specific Oligonucleotide primers (wild type and mutant nucleotides in capital letter, bold) were designed to screen mutant from non-mutant colonies after mutagenesis. ASO-forward primers were used in combination with pmir_seq_R. Sequencing primers: pmir_seq_F and pmir_seq_R primers were designed on the plasmid sequence and they were employed for post-cloning screening and sequencing check.

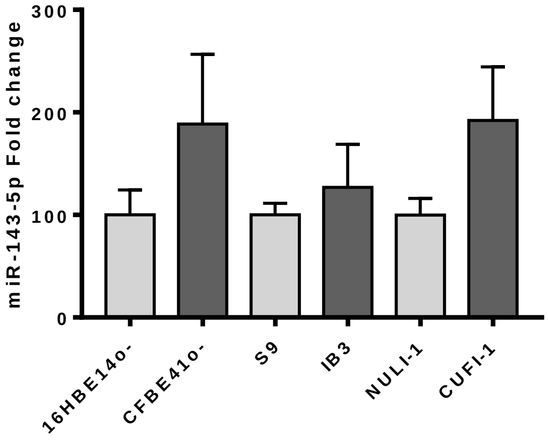

Figure S1. Relative expression of miR-143-5p in matched pairs of CF vs non–CF cell lines (i.e., CFBE41o$^-$ vs 16HBE14o$^-$, IB3 vs S9 and Cufi-1 vs Nuli-1, n = 6 for each cell line). Non-CF cells are reported as reference and set at 100%. Data are presented as mean ± SEM and were compared by Student's *t* test in the matched comparison. Although

miR-143-5p levels were trending upwards in all CF cell lines compared to their non-CF counterparts, none of the matched comparison reached the statistical significance.

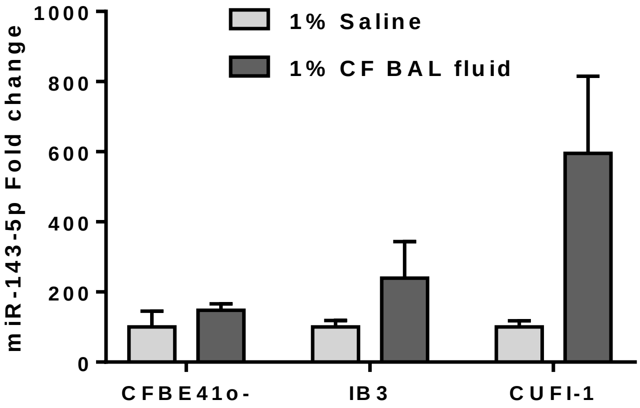

Figure S2. Relative expression of miR-143-5p in CF cell lines treated with CF BAL fluid vs vehicle control (saline solution) in three independent experiments (in duplicates). Control-treated samples are reported as reference and set at 100%. Data are presented as mean ± SEM and were compared by Student's *t* test within each cell line. Although we observed an increasing trend for miR-143-5p expression in all cell lines following treatment with CF BAL fluid compared to their control-treated samples, none of the comparisons reached the statistical significance.

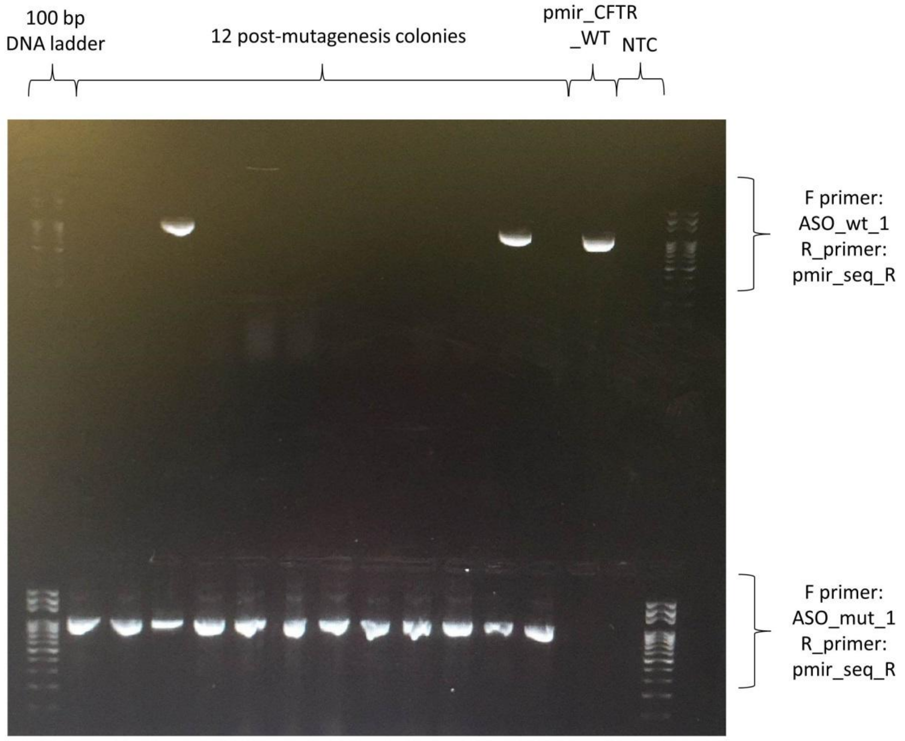

Figure S3. Example of post-mutagenesis screening performed with colony ASO-PCR: after post-mutagenesis transformation, bacterial colonies were picked from the plate and used directly as template for the ASO-PCR. ASO-forward primers were used in combination with the external primer pmir_seq_R. The amplicon product (1079 bp) was checked on a 2% agarose gel, together with a positive control (pmir_CFTR_wt, it should be amplified only in the mix containing the ASO_wt_1 primers) and a no template control (NTC). In this example, 10 out of 12 colonies were correctly transformed with the pmir_CFTR_mut1 plasmid.
